# Supplementary material for: Association of Serum Oxysterols with Cholesterol Metabolism Markers and Clinical Factors in Patients with Coronary Artery Disease: A Covariance Structure Analysis
Source: Nutrients. 2023 Jun 30;15(13):2997. doi: 10.3390/nu15132997 (PMC10346963; doi:10.3390/nu15132997)
Supplement: Supplementary file 1 [file nutrients-15-02997-s001.zip › nutrients-2453197-supplementary.pdf]

Supplementary Table S1. Result of the path model 1.

Clinical factor			Estimated value	Standard error	Statistic value	P value	Standardized estimates
Cholesterol absorption	←	Age	-0.019	0.081	-0.235	0.815	-0.018
	←	Male	-0.012	0.193	-0.061	0.951	-0.005
	←	Hypertension	-0.202	0.169	-1.192	0.233	-0.088
	←	Diabetes	-0.023	0.152	-0.153	0.879	-0.011
	←	Dyslipidemia	0.554	0.252	2.197	0.028	0.163
	←	Smoking	-0.033	0.173	-0.188	0.851	-0.014
	←	Statin usage	0.379	0.159	2.379	0.017	0.175
	←	hs-CRP	-0.123	0.079	-1.565	0.118	-0.129
Cholesterol synthesis	←	Age	-0.128	0.082	-1.560	0.119	-0.117
	←	Male	-0.239	0.195	1.222	0.222	0.091
	←	Hypertension	-0.286	0.171	-1.671	0.095	-0.12
	←	Diabetes	-0.137	0.154	-0.887	0.375	-0.063
	←	Dyslipidemia	0.288	0.253	1.139	0.255	0.082
	←	Smoking	-0.137	0.175	-0.784	0.433	-0.058
	←	Statin usage	0.588	0.162	3.626	<0.001	0.261
	←	hs-CRP	-0.113	0.079	-1.428	0.153	-0.114
Campesterol	←	Cholesterol absorption	0.113	0.030	3.726	<0.001	0.273
TRIOL	←		0.4	0.054	7.398	<0.001	0.542
4β-OHC	←		0.566	0.051	11.034	<0.001	0.958
24-OH-C	←		0.12	0.051	2.336	0.019	0.153
25-OH-C	←		0.294	0.053	5.589	<0.001	0.355
7α-OH-C	←		0.124	0.039	3.159	0.002	0.196
7β-OH-C	←		0.208	0.045	4.615	<0.001	0.277
7-keto-C	←		0.053	0.043	1.238	0.216	0.07
Lathosterol	←		-0.019	0.014	-1.383	0.167	-0.099
Campesterol	←	Cholesterol synthesis	0.005	0.029	0.187	0.852	0.013
24-OH-C	←		0.459	0.049	9.447	<0.001	0.609
25-OH-C	←		0.529	0.046	11.421	<0.001	0.665
27-OH-C	←		0.071	0.022	3.28	0.001	0.236
7α-OH-C	←		0.436	0.036	11.948	<0.001	0.72
7β-OH-C	←		0.561	0.041	13.733	<0.001	0.775
7-keto-C	←		0.658	0.041	15.984	<0.001	0.897
β-EPOX	←		0.443	0.042	10.579	<0.001	0.678
Lathosterol	←		0.058	0.013	4.347	<0.001	0.312
Age	↔	Male	-0.131	0.03	-4.346	<0.001	-0.318
	↔	Hypertension	0.08	0.032	2.476	0.013	0.175
	↔	Diabetes	-0.004	0.035	-0.124	0.902	-0.009

	↔	Dyslipidemia	0.0001	0.021	0.019	0.985	0.001
	↔	Smoking	-0.149	0.034	-4.416	<0.001	-0.323
	↔	Statin usage	-0.011	0.034	-0.34	0.734	-0.024
	↔	hs-CRP	0.058	0.084	0.691	0.489	0.053
Male	↔	Hypertension	-0.021	0.013	-1.562	0.118	-0.109
	↔	Diabetes	0.005	0.014	0.366	0.714	0.026
	↔	Dyslipidemia	-0.018	0.009	-2.042	0.041	-0.144
	↔	Smoking	0.048	0.014	3.497	<0.001	0.251
	↔	Statin usage	0.011	0.014	0.785	0.432	0.055
	↔	hs-CRP	-0.053	0.035	-1.505	0.132	-0.116
Hypertension	↔	Diabetes	0.046	0.016	2.862	0.004	0.204
	↔	Dyslipidemia	0.017	0.01	1.723	0.085	0.121
	↔	Smoking	-0.02	0.015	-1.356	0.175	-0.095
	↔	Statin usage	-0.013	0.015	-0.845	0.398	-0.059
	↔	hs-CRP	0.06	0.039	1.549	0.121	0.12
Diabetes	↔	Dyslipidemia	0.006	0.011	0.59	0.555	0.041
	↔	Smoking	0.019	0.016	1.201	0.23	0.084
	↔	Statin usage	0.02	0.017	1.17	0.242	0.082
	↔	hs-CRP	0.075	0.042	1.759	0.078	0.136
Dyslipidemia	↔	Smoking	0.014	0.01	1.358	0.175	0.095
	↔	Statin usage	0.009	0.01	0.844	0.399	0.059
	↔	hs-CRP	0.064	0.026	2.417	0.016	0.188
Smoking	↔	Statin usage	0.028	0.016	1.788	0.074	0.126
	↔	hs-CRP	0.03	0.039	0.763	0.445	0.059

hs-CRP=high sensitivity C-reactive protein, TRIOL=cholestan-3β,5α,6β-triol, 4β-OH-C=4β-hydroxy-cholesterol, 24-OH-C=24-hydroxy-cholesterol 7-keto-C=7-keto-cholesterol, β-epoxy-C=β-epoxy-cholesterol.

Supplementary Table S2. Result of the path model 2.

Clinical factor			Estimated value	Standard error	Statistic value	P value	Standardized estimates
Cholesterol absorption	←	Age	-0.078	0.079	-0.989	0.323	-0.074
	←	BMI	-0.026	0.021	-1.238	0.216	-0.088
	←	Hypertension	-0.166	0.167	-0.993	0.321	-0.072
	←	Diabetes	-0.021	0.147	-0.143	0.886	-0.01
	←	Dyslipidemia	0.569	0.24	2.367	0.018	0.167
	←	ACS	0.026	0.159	0.161	0.872	0.012
	←	Statin usage	0.362	0.151	2.399	0.016	0.167
	←	BNP	0.128	0.069	1.854	0.064	0.149
Cholesterol synthesis	←	Age	-0.112	0.082	-1.355	0.175	-0.103
	←	BMI	0.021	0.022	0.939	0.348	0.068
	←	Hypertension	-0.317	0.175	-1.809	0.07	-0.134
	←	Diabetes	-0.173	0.155	-1.122	0.262	-0.08
	←	Dyslipidemia	0.104	0.248	0.417	0.677	0.03
	←	ACS	0.103	0.166	0.621	0.534	0.047
	←	Statin usage	0.632	0.159	3.963	<.001	0.283
	←	BNP	-0.036	0.072	-0.506	0.613	-0.041
Campesterol	←	Cholesterol absorption	0.109	0.029	3.710	<.001	0.525
TRIOL	←		0.387	0.053	7.257	<.001	0.263
4β-OHC	←		0.588	0.053	11.162	<.001	0.996
24-OH-C	←		0.12	0.049	2.421	0.015	0.154
25-OH-C	←		0.283	0.051	5.522	<.001	0.345
7α-OH-C	←		0.117	0.038	3.104	0.002	0.187
7β-OH-C	←		0.204	0.044	4.627	<.001	0.272
7-keto-C	←		0.052	0.042	1.263	0.207	0.069
Lathosterol	←		-0.018	0.013	-1.364	0.172	-0.094
Campesterol	←	Cholesterol synthesis	0.008	0.029	0.288	0.774	0.02
24-OH-C	←		0.463	0.049	9.514	<.001	0.611
25-OH-C	←		0.54	0.046	11.64	<.001	0.676
27-OH-C	←		0.071	0.022	3.259	0.001	0.234
7α-OH-C	←		0.444	0.037	12.141	<.001	0.73
7β-OH-C	←		0.569	0.041	13.945	<.001	0.784
7-keto-C	←		0.662	0.041	16.038	<.001	0.897
β-EPOX	←		0.443	0.042	10.51	<.001	0.672

Lathosterol	←		0.059	0.013	4.354	<.001	0.31
Age	↔	BMI	-0.475	0.245	-1.943	0.052	-0.137
	↔	Hypertension	0.08	0.032	2.476	0.013	0.175
	↔	Diabetes	-0.004	0.035	-0.124	0.902	-0.009
	↔	Dyslipidemia	0.0001	0.021	0.019	0.985	0.001
	↔	ACS	-0.068	0.034	-2.011	0.044	-0.142
	↔	Statin usage	-0.011	0.034	-0.34	0.734	0.024
	↔	BNP	0.386	0.093	4.14	<.001	0.317
BMI	↔	Hypertension	0.323	0.113	2.855	0.004	0.203
	↔	Diabetes	0.247	0.123	2.012	0.44	0.142
	↔	Dyslipidemia	0.18	0.076	2.364	0.018	0.167
	↔	ACS	-0.124	0.118	-1.044	0.297	-0.073
	↔	Statin usage	-0.094	0.118	-0.795	0.427	-0.055
	↔	BNP	-0.548	0.316	-1.738	0.082	-0.128
Hypertension	↔	Diabetes	0.046	0.016	2.862	0.004	0.204
	↔	Dyslipidemia	0.017	0.01	1.723	0.085	0.121
	↔	ACS	-0.047	0.016	-2.977	0.003	-0.212
	↔	Statin usage	-0.013	0.015	-0.845	0.398	-0.059
	↔	BNP	0.028	0.041	0.679	0.497	0.05
Diabetes	↔	Dyslipidemia	0.006	0.011	0.59	0.555	0.041
	↔	ACS	-0.048	0.017	-2.81	0.005	-0.2
	↔	Statin usage	0.02	0.017	1.17	0.242	0.082
	↔	BNP	-0.02	0.045	-0.453	0.65	-0.033
Dyslipidemia	↔	ACS	-0.008	0.01	-0.742	0.458	-0.052
	↔	Statin usage	0.009	0.01	0.844	0.399	0.059
	↔	BNP	-0.054	0.028	-1.932	0.053	-0.143
ACS	↔	Statin usage	0.021	0.016	1.29	0.197	0.09
	↔	BNP	0.129	0.045	2.904	0.004	0.218

ACS=Acute coronary syndrome, BNP=brain natriuretic peptide , TRIOL=cholestan-3β,5α,6β-triol, 4β-OH-C=4β-hydroxy-cholesterol, 24-OH-C=24-hydroxy-cholesterol 7-keto-C=7-keto-cholesterol, β-epoxy-C=β-epoxy-cholesterol.

Supplementary Figure S1. Proposed Path model 2

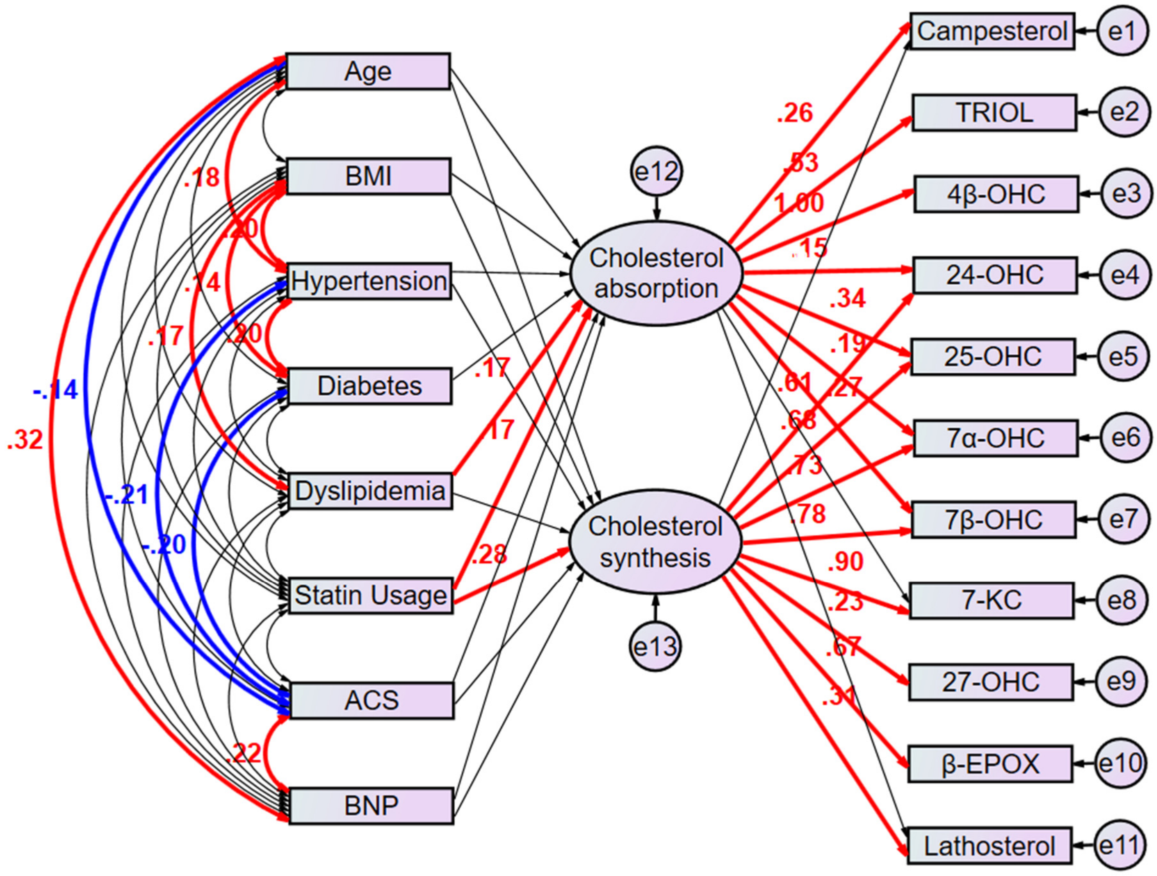

This path has a coefficient showing the standardized coefficient of regressing independent variables on the dependent variable of the relevant path. ACS=Acute coronary syndrome, BNP=brain natriuretic peptide, TRIOL=cholestan-3β,5α,6β-triol, 4β-OH-C=4β-hydroxy-cholesterol, 24-OH-C=24-hydroxy-cholesterol, 7-keto-C=7-keto-cholesterol, β-epoxy-C=β-epoxy-cholesterol.
